# An Atlas of the Cutaneous Nerves

**Published:** 1920-11-13

**Authors:** 


### An Atlas of the Cutaneous Nerves.

WE have received an advance copy of the above atlas, which has been carefully compiled by Mr. W. Ibbotson and is published by The Scientific Press, Ltd., Southampton Street, W.C. 2, at a price of 7s. 6d. net. A series of excellent diagrams in colour are designed to show in a simple manner the cutaneous nerve supply of the human body. Each has explanatory notes attached to it, and the latter give, in a concise manner, the exact origin of every nerve-trunk represented. Both the old and the "new" anatomical terminology are used. This atlas undoubtedly satisfies a long-felt want among minor medical text-books; it should be of great value to practitioners, students, and particularly to masseuses.

NATIONAL HOSPITAL FOR THE PARALYSED AND EPILEPTIC, QUEEN SQUARE, W.C.—Registrar, Miss M. A. Blandy, M.B.; Resident Medical Officer, Miss Mary R. Barkas, M.B.; Senior House Physician, A. G. Morison, M.B., Ch.B.; Junior House Physician, George A. Shepherd, M.B., Ch.B.

